# Strategic plan of the international association for child and adolescent psychiatry and allied professions (IACAPAP) for 2023–2026

**DOI:** 10.1186/s13034-023-00681-0

**Published:** 2023-12-13

**Authors:** Yewande O. Oshodi, Carmen M. Schroder, Luis Augusto Rohde, Daniel Fung

**Affiliations:** 1 Psychiatry Department, College of Medicine University of Lagos & Lagos University Teaching Hospital, Lagos, Nigeria; 2 Department of Child and Adolescent Psychiatry, Strasbourg University Hospitals, Institute for Cellular and Integrative Neurosciences, CNRS UPR 3212, University of Strasbourg, Strasbourg, France; 3 ADHD Outpatient Program & Developmental Psychiatry Program, Hospital de Clinicas de Porto Alegre, Federal University of Rio Grande do sul, Porto Alegre, Brazil; 4 Medical Council UniEduK, São Paulo, Brazil; 5 National Institute of Developmental Psychiatry & National Center for Innovation and Research in Mental Health, São Paulo, Brazil; 6 Yong Loo Lin Medical School and DUKE NUS Medical School, National University of Singapore. Institute of Mental Health, Singapore and the Lee Kong Chian School of Medicine, Nanyang Technological University, Singapore City, Singapore

The International Association for Child and Adolescent Psychiatry and Allied Professions (IACAPAP) has a strong commitment to advocating for the promotion of mental health and development of children and adolescents around the world through policy, practice and research. As a non-governmental organisation, its efforts are achieved largely through the contributions and the skill set of its membership. The IACAPAP membership is made up of more than 65 full or affiliate member associations, a huge amount of the worldwide regional or national Child and Adolescent Mental Health Associations. In addition, close to 300 individual members spread around the world, but mainly from countries where these national associations do not exist, take part of IACAPAP, making it a body with extensive outreach and huge potential for a global impact.

To achieve its set goals, the IACAPAP pursues a strategic plan which is revised regularly. Previous and the current Strategic plans are published online (here) and disseminated amongst members and partners as a blueprint to guide engagements and focus of the association. The current bureau started its tenure in December of 2022. Almost a year into leading this renewed body of child mental health experts, a look at the new strategic plan will give us insights into IACAPAP proposed directions for the upcoming years.

A critical part of IACAPAP's strategic plan is to develop a sustainable and scalable organizational structure. This includes improving internal and external communication, expanding its staff and volunteer base, monitoring strategic plans, and developing guidelines and standard operating procedures.

Highlights of the main goals within the 2023–2026 strategic plan include:

1. to catalyse joint initiatives with other organisations to improve child and adolescent mental health awareness and evidence-based care globally.
2. to support leadership and advocacy in child and adolescent mental health for national organisations and individual professionals.
3. to strengthen global training and professional development in child and adolescent mental health.
4. to create a global child and adolescent mental health ecosystem across cultures and languages.

In a bid to implement these goals, some key steps have already been taken including an innovative collaboration with the Child Mind Institute (CMI) based in New York. The CMI received a generous donation of USD 55 million in 2022 for developing Global Children’s Mental Health by Stavros Niarchos Foundation. The IACAPAP have secured a partnership program with the CMI to promote mental health for children and adolescents worldwide. Subsequently, a Memorandum of Understanding (MOU) with CMI was signed on the 13th of June 2023, framing the partnership between the IACAPAP and CMI on some key initiatives that are aligned with IACAPAP strategic plan, such as a global landscape mapping on child and adolescent mental health data. Additionally, a multicultural adaptation, implementation and training would also be set up to define web-based interventions that can be tested in different countries, mainly low- and middle-income countries. Finally, the collaboration will develop innovative global fellowship programs in child and adolescent mental health (CAMH). The already existing fellowships and study groups in IACAPAP, such as the Donald Cohen Fellowship Program will bring their wealth of experience to support the planning and development of this global fellowship program with CMI.

The support of leadership and advocacy in CAMH for national organisations and individual professionals also holds a position of importance in IACAPAP’s new strategic plan. The association offers membership benefits around access to training and learning resources via its member site, and this remains a priority to the executive council. A new members’ corner allows member associations now to announce their programs/ events (further encouraging growth of all member associations). In addition, since global training and development is central to its strategic goals, IACAPAP continues to provide well attended IACAPAP lunch and learn regional webinars, MOOCs and programs by the late Henrikje Klasen iCAMH. Improvements to these outlets include a specific focus on needs of the allied professional groups in upcoming webinars of 2024, as well as additional efforts to further extend its reach globally.

One major educational resource for child and adolescent psychiatrists and allied professions worldwide is the freely accessible IACAPAP e-textbook. A new revised and extended edition with a more attractive format for readers is currently underway, and new strategic partnerships and funding has been secured to enhance its visibility and access globally. As always, IACAPAP continue relying in Child and Adolescent Mental Health as its primary outlet for scientific relevant content in the area.

Lastly, IACAPAP’s biannual congresses play a significant role in helping the IACAPAP achieve its goal of global impact and professional development. The congress is usually held in strategic locations that allow global attendance of its members. The IACAPAP Congress of 2024 will be held in Brazil, and the 2026 congress in Germany. These major meetings continue to be the melting pot for IACAPAP’s academic and professional networks made up of a rich and diverse spread of expertise in CAMH research and clinical practice.

Overall, this strategic plan for 2023–2026 reflects IACAPAP's commitment to further promote the mental health and development of children and adolescents globally. The association's goals are centred on collaboration, education, and advocacy in striving towards its vision of a world where all children grow up healthy, both emotionally and physically, realizing their full potential to contribute to their societies. Exciting days lie ahead, and the bureau looks forward to growing an even stronger and impactful IACAPAP built on the firmly established foundation its predecessors have put in place.

## Data Availability

Not applicable.

